# Prevalence and caries-related risk factors in schoolchildren of 12- and 15-year-old: a cross-sectional study

**DOI:** 10.1186/s12903-019-0806-5

**Published:** 2019-06-18

**Authors:** N. Obregón-Rodríguez, P. Fernández-Riveiro, M. Piñeiro-Lamas, E. Smyth-Chamosa, A. Montes-Martínez, M. M. Suárez-Cunqueiro

**Affiliations:** 1 Department of Surgery and Medical Surgical Specialties, Medicine and Dentistry School, University of Santiago de Compostela, Santiago de Compostela, Spain; 2 Department of Psychiatry, Radiology and Public Health, University of Santiago de Compostela, Santiago de Compostela, Spain; 3 CIBER of Epidemiology and Public Health, CIBERESP, Santiago de Compostela, Spain; 4 Health Research Institute of Santiago de Compostela (IDIS), Travesía da Choupana, 15706 Santiago de Compostela, A Coruña Spain

**Keywords:** Dental caries, Risk factors, Toothbrushing, Oral hygiene, Schoolchildren, Adolescents, Dental plaque index

## Abstract

**Background:**

To assess the prevalence and severity of caries in 12- and 15-year-old schoolchildren, and to analyse the related risk factors.

**Methods:**

We conducted a cross-sectional study on a random sample of 1843 schoolchildren aged 12 and 15 from Galicia (northwest of Spain). Self-administered questionnaire and dental clinical examination were performed to obtain information about oral health habits, dental caries and oral hygiene. A logistic regression model including dental-caries-related variables was generated for each age group.

**Results:**

The respective findings for 12- and 15-years-old were as follows: decayed, missing, filled teeth index both for permanent and temporary dentition (DMFT/dmft) of 0.89 (95% CI, 0.87–0.91) and 1.38 (95% CI, 1.33–1.43), respectively; caries prevalence 39.6% (95% CI, 36.3–42.9) and 51.7% (95% CI, 48.0–55.4), respectively. In the 12-year-old group, individuals who occasionally, never or hardly ever brushed their teeth had higher values of caries (OR = 1.83, 95% CI 1.07–3.15, and OR = 9.14, 95% CI1.63–51.17, respectively). Also, the presence of plaque on more than 1/3 gingival was statistically associated with an increase of caries (OR = 2.03; 95% CI, 1.11–3.70), and living in a rural environment was a risk factor (OR = 1.3; 95% CI,1.02–1.80). In the 15-year-old group, higher caries risk was found when brushing was performed once a day (OR = 1.61; 95% CI,1.03–2.50), and among individuals who visited private clinics (OR = 1.77; 95% CI, 1.17–2.66), while electric toothbrush was associated with a lower caries risk (OR = 0.50; 95% CI, 0.29–0.86).

**Conclusions:**

This study revealed that risk factors of dental caries showed differences in schoolchildren of 12- and 15-year-old. Strongest evidence related to caries in 12-year-old group were found in frequency of toothbrushing and dental plaque. In 15-year old group, electric toothbrush, time since the last visit to the dentist and type of dental care (public/private) had a stronger association with dental caries. Caries prevalence and mean DMFT/dmft increased from 12- to 15-year-old, in spite of improvement in oral hygiene at the age of 15.

**Electronic supplementary material:**

The online version of this article (10.1186/s12903-019-0806-5) contains supplementary material, which is available to authorized users.

## Background

Caries is a multifactorial disease biofilm mediated. Among other reasons, its importance is due to the fact that it is one of the most prevalent diseases worldwide. Its prevalence among children and adolescents living in developed countries has decreased significantly in recent decades, increasing the number of caries-free subjects. Even so, and despite its being largely preventable, the disease remains a major public health problem in developed and developing countries because of the increase in consumption of sugary substances, poor oral hygiene practices, and inadequate use of dental services [1–4].

Galicia is a Regional Administration in the northwest of Spain and its surface is 5.9% of the country total surface area. An improvement in dental health was observed in Galician schoolchildren during the period 1995–2005 with a remarkable reduction in decayed, missing and filled teeth index (DMFT > 0) among 12-year-olds (from 64.2% in 1995 to 52.7% in 2005) [5].

12- and 15-year-old adolescents represent a very important study group in epidemiological surveys of caries due to several reasons: the easy access to this population at school, the final stage of permanent teeth eruption (three molars excluded) and the beginning of self-made decisions about diet and hygiene [6]. Accordingly, 15-year-olds are used as a reference group to study the trend in and severity of caries [2].

In addition to local factors, the aetiology of this disease includes factors which act both at an individual and at a community level. Numerous studies have linked caries to oral hygiene, regular use of fluoride, dietary habits, dental care and health policies, among other factors, so that these have been established as important risk factors of caries appearance and progression [7–12]. Knowledge of these risk factors is essential in order to know the population’s oral health status and how it could be improved, and also for planning effective dental public health policies.

The Spanish Society of Epidemiology and Oral Public Health (SESPO) and the National Dental Association defined basic goals for oral health in Spain by 2015–2020 [13, 14], following the goals proposed by the WHO Oral Health Guidance for 2020 [15]: mean DMFT in 12 years old adolescents should be ≤1.0, restoration index should be ≥60% at 12-year-old and ≥ 65% at 15-year-old; Significant Caries Index (Sic) should be ≤3 at 12-year-old; the prevalence of caries-free population should be ≥68% and ≥ 57% at 12- and 15-years-old, respectively; and more than 91% of the individuals should brush their teeth more than once a day with fluoride paste [14–16]. At the same time, in 2010, it was launched the Alliance for a Cavity-Free Future (ACFF), that is a global not-for-profit organisation dedicated to promote integrated clinical and public health action in order to stop the initiation and progression of dental caries. The work of the AFCFF Globally is centered around achieving four main goals: 1) every child born after 2026 should stay caries-free during their lifetime; 2) dental schools and dental associations should have accepted the caries preventive philosophy management; 3) ACFF will work to achieve a reduction in caries inequality in the context of oral and general health; 4) by 2020, regional members of the ACFF should have integrated, comprehensive and locally appropriate caries prevention and management systems and monitoring approaches developed and in place [17].

Within this context, the aim of this study was to assess the prevalence and severity of caries in 12- and 15-year-old schoolchildren, and to analyse related risk factors.

## Methods

### Study design and population

An oral health epidemiological survey of random samples of schoolchildren aged 12 and 15 years old from the northwest of Spain (Galicia region) was performed following international standards stablished by WHO for this type of study [6]. The study was descriptive cross-sectional and national-level systematic one.

The target size population of this study was over 23,500 pupils of 12-years-old and 20,000 of 15-years-old, from a total of 1067 classrooms of 485 schools of secondary education. This information was obtained according to the registered schools and pupils supplied by the Regional Administration. To select the sample, data from the latest oral health survey in Galicia was used. We calculated a random sample from all secondary schools (public, non-public), stratified by province and size of town (≥20,000 inhabitants or < 20,000 inhabitants). A classroom was randomly selected in each school. The sample included 60 schools; 60 classrooms of schoolchildren aged 12 and 60 classrooms of 15-year-old schoolchildren. All the individuals of every classroom were included in the sample aged 12. In the sample aged 15, we randomly selected 12 schoolchildren from each selected classroom. Sample size was 1266 individuals aged 12 and 720 individuals aged 15. Sample size was obtained with 95% confidence level and an absolute error of 3,5% [5]. Sample size was increased 10% to compensate expected missing pupils and to correct design effect that was assumed as 1,5.

To achieve greater representativeness, the sample was weighted, taking into account the method of sample selection, and each subject then being reweighted, to adjust the distribution of the sample to the population of Galicia of that age, gender and province.

Five working teams (a dentist and an hygienist) performed the oral examinations and collected the data from the questionnaires. The dental examination was performed by a dentist, using a dental mirror and a WHO periodontal probe, following the WHO recommendations for oral health surveys [6]. Caries was defined as a cavitated lesion. Dental plaque index was monitored visually and with the help of the WHO CPI probe to determine the presence/ absence of dental plaque on the buccal surfaces of the explored teeth (16, 11, 26, 36, 31, 46), being clasified as dental plaque-free, dental plaque on the gingival border, on 1/3 gingival or dental plaque on more than 1/3 gingival. The questionnaires were distributed by the oral hygienist to all the selected students in the classroom and explained to them how to complete it, solving any doubt they could have about the questions. All working teams were previously trained for the purpose of standardising the survey protocols. A preliminary calibration was carried out, in which the working groups were trained with different model- and student-based examples until the same criteria were obtained for performing the examinations. During the study, 10% of the sample was explored twice, once by the working team and another the external operator or “faithful” calibrator to determine the diagnostic concordance among all the examiners. Cohen’s Kappa index was calculated to determine both interobserver and intraobserver agreement. We calculated the unweighted Kappa index (KU) and the weighted Kappa index (Kw) with quadratic weights. The KU considers that all disagreements have the same importance, while the Kw gives greater reliability when the disagreements between the evaluators are small compared to when they are large. Interobserver and intraobserver Kappa index indicated noticeably good agreement (Additional file 1: Table S1 and Additional file 2: Table S2).

### Definition of variables

Two variables were defined to study caries involvement: the decayed, missing, filled permament and temporary teeth index (DMFT/dmft) and caries prevalence (DMFT/dmft > 0). The DMFT/dmft index was recorded according to WHO guidelines [6]. The WHO uses this index as the indicator to compare dental health status among different populations. Caries prevalence is the proportion of individuals with caries, being classified as affected (DMFT/dmft > 0) or not affected (DMFT/dmft = 0). Caries was defined as a cavitated lesion.

Other indicators were calculated, such as the SiC (mean DMFT for the third of the population with the highest levels of caries), Restoration Index (percentage of filled teeth within total caries experience index), Dental Health Index (caries-free teeth minus DMFT, divided by 28), and Functional Dental Index (sum of caries-free and filled teeth divided by 28) were calculated [6].

The following variables were analysed in order to study their association with dental caries: toothbrushing; type of toothbrush; starting age of toothbrushing; dental hygiene (dental plaque index); fluoride; dental floss; cariogenic diet; private or public dental care; and time since last visit to the dentist. The socio-demographic variables analysed were age, sex and residence. These variables and their categories are shown in Table 1.

**Table 1:** Distribution of variables in the sample of Galician schoolchildren, by age group

Variables		Prevalence				
		Age 12 years		Age 15 years		*p*-value
		n	Percentage (95% CI)	n	Percentage (95% CI)	
Sex	Male	501	47.8% [44.5–51.2]	339	43.2% [39.6–46.9]	0.0706
	Female	544	52.2% [48.8–55.5]	444	56.8% [53.1–60.4]	
Residence	Urban area	605	53.5% [50.1–56.8]	488	54.6% [50.9–58.3]	0.6482
	Rural area	440	46.5% [43.2–49.9]	295	45.4% [41.7–49.1]	
Frequency of toothbrushing	More than once a day	698	65.8% [62.5–68.9]	611	78.0% [74.7–80.9]	< 0.0001
	Once a day	226	22.5% [19.8–25.5]	134	16.8% [14.2–19.7]	
	Less than once a day	21	2.0% [1.2–3.2]	15	1.9% [1.2–3.2]	
	Occasionally	86	8.5% [6.8–10.6]	20	3.2% [2.0–5.1]	
	Never or hardly ever	10	1.2% [0.6–2.4]	1	0.1% [0.0–0.6]	
Type of toothbrush	Manual	874	85.3% [82.7–87.6]	705	92.0% [89.7–93.8]	0.0008
	Electric	294	33.9% [30.5–37.4]	167	23.9% [20.7–27.4]	
	Both	133	12.7% [10.7–15.1]	81	10.4% [8.5–12.8]	
Toothbrushing starting age	Mean ± SD	3.82 ± 1.55	- [3.72–3.92]	3.94 ± 1.81	- [3.81–4.07]	0.1775^c^
Dental hygiene^a^	Absence of dental plaque	218	19.9% [17.3–22.7]	279	36.2% [32.7–39.9]	< 0.0001
	Plaque on gingival border	479	46.1% [42.7–49.4]	350	44.9% [41.2–48.6]	
	Plaque on 1/3 gingival	261	25.5% [22.7–28.6]	128	15.8% [13.2–18.7]	
	Plaque on > 1/3 gingival	86	8.6% [6.8–10.7]	26	3.1% [2.1–4.7]	
Fluorides	Yes	954	91.3% [89.2–93.1]	709	90.5% [88.0–92.6]	< 0.0001
	No	91	8.7% [6.9–10.8]	74	9.5% [7.4–12.0]	
Dental floss	Yes	159	18.6% [15.9–21.7]	127	19.2% [16.3–22.5]	0.7942
	No	707	81.4% [78.3–84.1]	552	80.8% [77.5–83.7]	
Dietary habits^b^	Cariogenic diet	983	94.4% [92.6–95.7]	410	52.1% [48.4–55.7]	< 0.0001
	Non-cariogenic diet	62	5.6% [4.3–7.4]	373	47.9% [44.3–51.6]	
Type of dental care	Public dental health services	341	33.3% [30.2–36.6]	163	20.3% [17.5–23.4]	< 0.0001
	Private dental clinics	686	66.7% [63.4–69.8]	610	79.7% [76.6–82.5]	
Time since last visit to the dentist	Less than 1 month	307	30.5% [27.4–33.7]	216	28.1% [24.9–31.6]	0.0769
	1–3 months	241	24.6% [21.8–27.7]	178	23.0% [20.1–26.3]	
	4–5 months	246	24.0% [21.3–27.1]	167	21.8% [18.9–25.1]	
	More than 6 months	208	20.2% [17.6–23.0]	205	26.2% [23.1–29.6]	
	Never	8	0.7% [0.3–1.5]	7	0.8% [0.4–1.7]	
Caries prevalence	Yes	409	39.6% [36.3–42.9]	385	51.7% [48.0–55.4]	< 0.0001
	No	636	60.4% [57.1–63.7]	398	48.3% [44.6–52.0]	

Statistical evaluation using the Chi-square test with 2nd-order Rao-Scott correction for differences by age group

^a^Dental plaque of explored teeth (16, 11, 26, 36, 31, 46): 0 = Dental plaque-free; 1 = Dental plaque on gingival border; 2 = Dental plaque on 1/3 gingival; 3 = Dental plaque on more than 1/3 gingival

^b^Question: *Which of these foods have you eaten the last week?* a) Candies b) Chewing gums and jelly beans c) Soft drinks and processed juices d) Chocolate e) Popcorn and crackers f) Pastries and cakes g) Ice creams h) Others (to specify). To mark the frequency of consumption of each food: None/ 3 times a week or less/ 4–6 times a week/ Everyday

^c^Statistical evaluation using the Student’s t-test for the difference of means by age group

### Ethical considerations

Approval by the Galician Clinical Research Ethics Committee was not required, since the study formed part of a larger project classified by the Galician Regional Administration (*Xunta de Galicia*) as an evaluation of services. Participation was voluntary. An information sheet was circulated to and informed written consent was obtained from the families and/or legal guardians of the study participants. To ensure confidentiality, a numerical code was used for each individual.

### Statistical analysis

All the statistical analysis have been carried out taking into account that the data have been obtained through a complex sampling design. The characteristics of the sample were described by frequency distribution, with the means of the DMFT/dmft index and the different caries indicators being calculated for each age group. Differences by age group were evaluated using the Chi-square test with 2nd-order Rao-Scott correction for the qualitative variables and the Student’s t-test was used to evaluate the difference of means for the continuous variables. A complex sample analysis was performed for binomial dependent variables (caries prevalence) to calculate the Odds Ratios (ORs) and their adjusted confidence intervals (CIs). In order to construct the models, we first performed a bivariate analysis with the exposure variables and potential confounding variables; we then fitted a multivariate logistic regression model, including those independent variables that were statistically significantly lower than 0.2 in the bivariate analysis. Independent variables with a higher level of statistical significance were eliminated from this model, provided that the coefficients of the main exposure variables were not changed by more than 10% and the Akaike Criterion improved. All *p*-values were two-sided, with *p*-values of 0.05 or less being deemed to be statistically significant. The R Survey Package (version 2.3.0) was used to perform all the statistical analysis.

## Results

The final sample consisted of 1843 individuals, 1055 aged 12, and 788 aged 15 years. Due to absence from school on the day of the survey, there were 10 losses in the former and 5 in the latter group, resulting in 1045 and 783 individuals respectively. After being weighted, this sample represented 17,202 schoolchildren aged 12 and 5512 aged 15.

A total of 65.8% of individuals aged 12 reported brushing their teeth more than once a day, a percentage that increased in the 15-year age group. While the use of fluoride in toothpastes, rinses or gels was 91.3 and 90.5% for 12- and 15-year-olds respectively, the use of dental floss was below 20% in both groups. The mean of reported toothbrushing starting age was 3.88 year-oldds (SD ± 1.67), without significant differences between age groups. With respect to dietary habits, 94.4% of the sample aged 12 and 52.1% of the sample aged 15 reported a cariogenic diet, with statistically significant differences. Males showed in both age groups, the highest values 96.2 and 55% of consumption respectively.

The proportion of schoolchildren who attended the dentist within the last 6 months exceeded 70% in both age groups. Private dentists had higher percentages of schoolchildren than the public dental health service (provided by SERGAS) in both age groups.

Caries prevalence increased with age, from 39.6% at 12 years to 51.7% at 15 years. Although there was a statistically significant difference between age groups, there was no evidence of any significant gender-related differences. Caries prevalence was approximately 6% higher among children living in rural areas but these differences were not statistically significant.

The DMFT/dmft index was higher in 15- than in 12-year-old group, with means of 1.38 (SD ± 1.87) and 0.82 (SD ± 1.46) respectively, partly due to the higher proportion of filled teeth (1.22 ± 1.75). The mean number of decayed teeth was slightly higher at age 12 (0.22 ± 0.75 vs. 0.15 ± 0.52, respectively), as shown in Table 2. The restoration index was 87.13 (SD ± 29.98) at 15, 14% higher than at 12-year-old group. The SiC index was also higher for 15-year-old (3.44 ± 1.75 vs. 2.46 ± 1.56), with statistically significant differences. No significant gender-related differences were found in any of these indicators. The overall proportion of affectation was also higher in the 15-year-old group, 5.01%, with this being higher in girls (5.4%) than in boys (4.5%). Statistically significant differences between age groups were also found in both indicators. Other indicators of oral health status, such as the Functional Dental Index and Dental Health Index, displayed similar values for both groups.

**Table 2:** Indicators of dental caries by age group: mean, standard deviation (SD) and 95% confidence intervals, by age group

Indicator		Age 12 years	Age 15 years	*p*-value
Caries-free teeth^a^	Mean ± SD	25.48 ± 2.42	26.23 ± 2.12	< 0.0001
	95% CI	[25.44–25.52]	[26.18–26.29]	
Decayed teeth^a^	Mean ± SD	0.22 ± 0.75	0.15 ± 0.52	0.0252
	95% CI	[0.21–0.23]	[0.13–0.16]	
Missing teeth^a^	Mean ± SD	0.05 ± 0.32	0.22 ± 0.80	< 0.0001
	95% CI	[0.04–0.05]	[0.20–0.24]	
Filled teeth^a^	Mean ± SD	0.66 ± 1.19	1.22 ± 1.75	< 0.0001
	95% CI	[0.64–0.68]	[1.17–1.26]	
DMFT/dmft Index^b^	Mean ± SD	0.89 ± 1.46	1.38 ± 1.87	< 0.0001
	95% CI	[0.87–0.91]	[1.33–1.43]	
SiC Index^c^	Mean ± SD	2.46 ± 1.56	3.44 ± 1.75	< 0.0001
	95% CI	[2.42–2.50]	[3.37–3.52]	
Restoration Index (%)^d^	Mean ± SD	73.29 ± 39.42	87.13 ± 29.98	< 0.0001
	95% CI	[72.36–74.23]	[86.03–88.24]	
Proportion of affectation (%)^e^	Mean ± SD	3.42 ± 5.64	5.01 ± 6.79	< 0.0001
	95% CI	[3.34–3.50]	[4.83–5.19]	

Statistical evaluation using the Student’s t-test for the difference of means by age group

^a^Total number (temporary + permanent) of caries-free, decayed, missing and filled teeth

^b^Average decayed, missing and filled teeth per child, including temporary and permanent teeth

^c^Brathall’s SiC Index. DMFT for the third of the population with the highest caries levels (higher DMFT Index)

^d^Ratio between filled teeth and DMFT/dmft Index, multiplied by 100

^e^Ratio between DMFT/dmft Index and total examined teeth, multiplied by 100

### Multivariate regression analyses

Tables 3 and 4 show the results of the logistic regression analysis for variables associated with the presence of decayed teeth in permanent and temporary teeth in both age groups. Among 12-year-olds, those who never or hardly ever brushed their teeth had a higher caries risk (OR = 9.14; 95% CI, 1.63–51.17) than those who brushed their teeth more than once a day. Poor dental hygiene showed strong evidence of caries risk, with statistically significant differences for dental plaque on 1/3 gingival (OR = 1.55; 95% CI, 1.01–3.15) or more than 1/3 (OR = 2.03; 95% CI, 1.11-a3.70). Patients living in rural areas registered a higher caries risk than did those residing in urban areas (OR = 1.53, 95% CI, 1.02–1.80). Type of dental care and cariogenic diet were not statistically significant in the multivariate analysis. Nevertheless, cariogenic diet showed twice the odds of having caries than non-cariogenic diet in the bivariate logistic regression model (OR = 2.21; 95% CI, 1.15–4.25). There have been observed statistically significant differences between public and private dental care for DMFT components. In private dental care, higher mean values of filling component were observed for both dentitions together (0.75 ± 0.006, 95% CI, 0.64–0.86) and for permanent dentition (0.68 ± 0.05, 95% CI, 0.58–0.79) than in public dental care, as shown in Table 5.

**Table 3:** Estimation of the association between the studied variables and caries prevalence in the 12-year-old group, using bivariate and multivariate logistic regression models

Variables	Bivariate model			Multivariate model		
	OR	95% CI	*p*-value	OR^a^	95% CI	*p*-value
Residence						
Urban area	1^b^			1^b^		
Rural area	1.31	(0.99–1.73)	0.0582	1.53	(1.02–1.80)	0.0380
Frequency of toothbrushing						
More than once a day	1^b^			1^b^		
Once a day	1.32	(0.94–1.85)	0.1063	1.27	(0.90–1.79)	0.1816
Less than once a day	1.54	(0.57–4.18)	0.3970	1.33	(0.48–3.65)	0.5863
Occasionally	1.86	(1.14–3.05)	0.0136	1.83	(1.07–3.15)	0.0287
Never or hardly ever	10.34	(1.96–54.39)	0.0059	9.14	(1.63–51.17)	0.0120
Dental hygiene						
Absence of dental plaque	1^b^			1^b^		
Plaque on gingival border	1.38	(0.94–2.03)	0.1020	1.27	(0.86–1.89)	0.2270
Plaque on 1/3 gingival	1.72	(1.12–2.63)	0.0132	1.55	(1.01–2.39)	0.0472
Plaque on more than 1/3 gingival	2.17	(1.23–3.84)	0.0077	2.03	(1.11–3.70)	0.0216
Type of dental care						
Public dental health services	1^b^			1^b^		
Private dental clinics	1.20	(0.89–1.61)	0.2316	1.31	(0.97–1.77)	0.0770
Dietary habits						
Non-cariogenic diet	1^b^			1^b^		
Cariogenic diet	2.21	(1.15–4.25)	0.0179	1.90	(0.97–3.72)	0.0626

^a^Adjusted for the effect of the other independent variables included in this table

^b^Reference group

**Table 4:** Estimation of the association between the studied variables and caries prevalence in the 15-year-old group, using bivariate and multivariate logistic regression models

Variables	Bivariate model			Multivariate model		
	OR	95% CI	*p-*value	OR^a^	95% CI	*p*-value
Frequency of toothbrushing						
More than one a day	1^b^			1^b^		
Once a day	1.64	(1.10–2.44)	0.0153	1.61	(1.03–2.50)	0.0351
Less than once a day	1.57	(0.54–4.58)	0.4102	1.62	(0.49–5.41)	0.4331
Occasionally	3.34	(1.05–10.66)	0.0417	3.50	(1.00–12.23)	0.0496
Never or hardly ever	–	–	–	–	–	–
Type of toothbrush						
Manual	1^b^			1^b^		
Electric	0.56	(0.33–0.93)	0.0248	0.50	(0.29–0.86)	0.0109
Both	0.60	(0.38–0.97)	0.0360	0.61	(0.37–0.99)	0.0471
Toothbrushing starting age	1.10	(1.02–1.21)	0.0140	1.09	(1.00–1.20)	0.0637
Dental hygiene						
Absence of dental plaque	1^b^			1^b^		
Plaque on gingival border	1.31	(0.98–1.83)	0.1096	1.39	(0.98–1.98)	0.0643
Plaque on 1/3 gingival	1.83	(1.16–2.90)	0.0098	1.57	(0.97–2.56)	0.0678
Plaque on more than 1/3 gingival	1.49	(0.65–3.44)	0.3455	1.19	(0.45–3.15)	0.7251
Time since last visit to the dentist						
Less than 1 month	1^b^			1^b^		
1–3 months	1.17	(0.77–1.78)	0.4675	1.21	(0.78–1.88)	0.4034
4–6 months	0.71	(0.46–1.09)	0.1157	0.71	(0.45–1.12)	0.1425
More than 6 months	0.62	(0.42–0.93)	0.0218	0.60	(0.39–0.93)	0.0232
Type of dental care						
Public dental health services	1^b^			1^b^		
Private dental clinics	1.77	(1.22–2.57)	0.0029	1.77	(1.17–2.66)	0.0068

^a^Adjusted for the effect of the other independent variables included in this table

^b^Reference group

**Table 5:** Indicators of dental caries by dental care type: mean, standard deviation (SD) and 95% confidence intervals at 12-year-old group

Indicator		Public dental health services	Private dental clinics	*p*-value
Caries-free teeth^a^	Mean ± SD	25.82 ± 0.14	25.33 ± 0.11	0.0049
	95% CI	[25.55–26.08]	[25.11–25.54]	
Decayed teeth^a^	Mean ± SD	0.28 ± 0.04	0.20 ± 0.04	0.1564
	95% CI	[0.20–0.37]	[0.13–0.27]	
Missing teeth^a^	Mean ± SD	0.03 ± 0.01	0.06 ± 0.02	0.1036
	95% CI	[0.01–0.05]	[0.03–0.09]	
Filled teeth^a^	Mean ± SD	0.48 ± 0.06	0.75 ± 0.06	0.0004
	95% CI	[0.37–0.58]	[0.64–0.86]	
Caries-free temporary teeth	Mean ± SD	1.20 ± 0.14	0.99 ± 0.09	0.1988
	95% CI	[0.92–1.47]	[0.82–1.16]	
Decayed temporary teeth	Mean ± SD	0.16 ± 0.03	0.08 ± 0.02	0.0254
	95% CI	[0.10–0.23]	[0.05–0.11]	
Filled temporary teeth	Mean ± SD	0.05 ± 0.02	0.07 ± 0.02	0.4356
	95% CI	[0.02–0.09]	[0.04–0.10]	
Caries-free permanent teeth	Mean ± SD	24.62 ± 0.23	24.34 ± 0.16	0.3098
	95% CI	[24.17–25.06]	[24.04–24.64]	
Decayed permanent teeth	Mean ± SD	0.12 ± 0.03	0.12 ± 0.03	0.9319
	95% CI	[0.06–0.17]	[0.06–0.19]	
Missing permanent teeth	Mean ± SD	0.01 ± 0.01	0.01 ± 0.01	0.4653
	95% CI	[0–0.02]	[0–0.01]	
Filled permanent teeth	Mean ± SD	0.42 ± 0.05	0.68 ± 0.05	0.0005
	95% CI	[0.32–0.52]	[0.58–0.79]	
DMFT/dmft^b^	Mean ± SD	0.77 ± 0.08	0.96 ± 0.07	0.0580
	95% CI	[0.62–0.92]	[0.83–1.10]	
DMFT^c^	Mean ± SD	0.55 ± 0.06	0.81 ± 0.07	0.0031
	95% CI	[0.43–0.67]	[0.68–0.94]	

Statistical evaluation using the Student’s t-test for the difference of means by age group

^a^Total number (temporary + permanent) of caries-free, decayed, missing and filled teeth

^b^Average decayed, missing and filled teeth per child, including temporary and permanent teeth

^c^Average decayed, missing and filled permanent teeth per child

At age 15, the multivariate analysis showed statistically significant differences for individuals who brushed their teeth once a day (OR = 1.61; 95% CI, 1.03–2.50) or ocasionally (OR = 3.50; 95% CI, 1.00–12.23). In terms of toothbrush type, electric brushes and a combination of manual and electric brushes were significant related to less caries prevalence (OR = 0.50; 95% CI, 0.29–0.89 and OR = 0.61; 95% CI, 0.37–0.99, respectively). Time since the last visit to the dentist was statistically significant when this time was greater than 6 months (OR = 0.60; 95% CI, 0.39–0.93), while caries history was higher among individuals who attended private clinics (OR = 1.77; 95% CI, 1.17–2.66). If we consider the DMFT components, the individuals who attended private dentists showed higher values of filling component for both dentitions (1.36 ± 0.08; 95% CI, 1.21–1.52) and for permanent dentition (1.35 ± 0.08; 95% CI, 1.20–1.51) than the schoolchildren who attended public dental health service. These results are shown in Table 6.

**Table 6:** Indicators of dental caries by dental care type: mean, standard deviation (SD) and 95% confidence intervals at 15-year-old group

Indicator		Public dental health services	Private dental clinics	*p*-value
Caries-free teeth^a^	Mean ± SD	26.90 ± 0.13	26.06 ± 0.10	< 0.0001
	95% CI	[26.65–27.15]	[25.86–26.23]	
Decayed teeth^a^	Mean ± SD	0.13 ± 0.04	0.14 ± 0.02	0.8320
	95% CI	[0.06–0.21]	[0.10–0.17]	
Missing teeth^a^	Mean ± SD	0.13 ± 0.05	0.25 ± 0.04	0.0503
	95% CI	[0.03–0.23]	[0.18–0.33]	
Filled teeth^a^	Mean ± SD	0.74 ± 0.10	1.36 ± 0.08	< 0.0001
	95% CI	[0.53–0.94]	[1.21–1.52]	
Caries-free temporary teeth	Mean ± SD	0.05 ± 0.02	0.06 ± 0.02	0.6097
	95% CI	[0–0.09]	[0.03–0.09]	
Decayed temporary teeth	Mean ± SD	0.01 ± 0.01	0.01 ± 0.00	0.7848
	95% CI	[0–0.01]	[0–0.01]	
Filled temporary teeth	Mean ± SD	0 ± 0	0 ± 0	0.0458
	95% CI	[0–0]	[0–0.01]	
Caries-free permanent teeth	Mean ± SD	26.86 ± 0.13	25.99 ± 0.10	< 0.0001
	95% CI	[26.60–27.11]	[25.80–26.17]	
Decayed permanent teeth	Mean ± SD	0.13 ± 0.04	0.14 ± 0.02	0.8563
	95% CI	[0.06–0.20]	[0.10–0.18]	
Missing permanent teeth	Mean ± SD	0.01 ± 0.01	0.02 ± 0.01	0.5073
	95% CI	[0–0.02]	[0–0.04]	
Filled permanent teeth	Mean ± SD	0.74 ± 0.10	1.35 ± 0.08	< 0.0001
	95% CI	[0.53–0.94]	[1.20–1.51]	
DMFT/dmft^b^	Mean ± SD	0.88 ± 0.11	1.52 ± 0.09	< 0.0001
	95% CI	[0.68–1.09]	[1.36–1.69]	
DMFT^c^	Mean ± SD	0.88 ± 0.11	1.51 ± 0.08	< 0.0001
	95% CI	[0.66–1.09]	[1.34–1.67]	

Statistical evaluation using the Student’s t-test for the difference of means by age group

^a^Total number (temporary + permanent) of caries-free, decayed, missing and filled teeth

^b^Average decayed, missing and filled teeth per child, including temporary and permanent teeth

^c^Average decayed, missing and filled permanent teeth per child

## Discussion

This study showed that risk factors of dental caries are not the same in both age groups. Although there are only 3 years of interval between both age groups, in this study individuals of 12 and 15-years-old must be analysed separately. Whereas rural area of residence, occasional, never or hardly ever toothbrushing and the presence of dental plaque on 1/3 gingival or more than 1/3 gingival showed a higher association with caries among 12-years-old, this was not the case at age 15. Among 15-years-old, toothbrushing once a day and private dental care showed higher association with caries, while the use of an electric toothbrush and a time lapse of more than 6 months since the last dental visit appeared to be related to lower caries levels. At age 15, an improvement in oral behaviour was in evidence. The study also showed an increase for caries prevalence and mean DMFT/dmft among 15-years-old due to the higher filling component. Regarding the SiC index and caries prevalence, our results showed inequalities among the population because higher levels of pathology are presented in a small part of the sample.

In this study, students with a toothbrushing frequency of more than once a day displayed lower caries levels than did individuals who reported a lower frequency, especially those who reported brushing their teeth ocasionally, never or hardly ever, a finding that suggests the effectiveness of toothbrushing in the prevention of caries, in line with the conclusion reached by other similar studies [1, 18–20]. The habit of toothbrushing once or more times per day stands at around 90% for both age groups. This finding is similar to the toothbrushing frequency from Spanish adolescents aged 12 and 15, who reported more than 90% brushing their teeth once or more times daily [16], with this being higher among women, a situation already described in the literature [10, 21]. According to a number of studies, oral hygiene is crucial for caries prevention, and behavioural changes in adolescence in relation to these factors may have a decisive influence on the oral status of adult patients [1, 2, 19]. This study showed at age 12, the presence of dental plaque on more than 1/3 gingival was associated with higher caries levels, suggesting that poor oral hygiene significantly increases caries risk. Some studies [2, 12, 22] also suggest a relationship between urban area of residence and adequate oral hygiene practicesand that makes us think that it would be appropiate to have other factors in mind, as high socio-economic level and educational attainment [12, 20]. In contrast to other studies [18], no association was found between dental caries and area of residence in 15-year-olds .

Our study found no significant relationship between the use of fluoride and caries prevalence. Rothen et al. [19] observed an increase in average caries in individuals who used fluoride products other than toothpaste, a fact that could not be confirmed in this study.

Regarding dietary habits, the consumption of sugary foods and sweet beverages is known to be associated with high caries rates and dental erosion [9, 23, 24]. In most studies [1, 10, 11, 24, 25], this increase is related to the frequency of sugar intake between meals. In our study, a high percentage of 12-years-old reported a cariogenic diet (94.4%), a situation which had improved by the age of 15 (50%). A statistically significant relationship was found between cariogenic diet and increased caries prevalence in the 12-year age group, in line with Gao et al. [23] who reported a strong relationship between consumption of sweets and DMFT/dmft values in younger children.

With respect to the type of dental care during the last visit to the dentist, caries prevalence was higher in both age groups who attended private clinics. Some studies [26–28] report that subjects with easy access to dental attendance and public dental health services are less likely to report oral impacts. In this study, the reasons why the patients surveyed went to one or another dental service are unknown. The higher caries prevalence among patients in the 15-year age group who visited private dentists might be due to coverages offered by the Galician dental health service (SERGAS) are limited to children aged 6 to 14-years-old; including only dental health education, topic fluorides, sealants and the filling of occlusal caries in permanent first molars [29].

Analysis of the interval since the last visit to the dentist showed small differences between age groups, with approximately 30% of schoolchildren reporting a dental visit less than 1 month previously. This figure was lower for the 15-year-old group, contrary to what is described by Aleksejūniene et al. [8], whose results indicate an increase in the positive attitude towards dental visits, with an increase in their regularity among older age groups. In general, a significant association between caries history and recent dental visits is widely accepted in the literature [11, 12], with children receiving dental attendance in recent months tending to have a higher DMFT. An interesting finding in our study is that at age 15, individuals who went to the dentist more than 6 months previously registered less caries history than those who went within the previous 3 months. In line with Crocombe et al’s findings [27], this might well indicate that many individuals visit their dentist more often for other reasons than prevention.

According to the WHO Database [30], most of the European countries have reduced the mean values of DMFT in 12-year-old adolescents in the last decades. Recent national surveys show mean DMFT of 0.4 in Denmark (2014), 0.5 in Germany (2014), 0.6 in Netherlands (2012), 0.7 in UK (2011), 0.7 in Spain (2014), and 0.8 in Sweden (2011), among others [30, 31]; and when compared to some studies conducted in Europe [1, 4, 7], mean DMFT values in our study have low values. On the other hand, countries such as Latvia (2.9 in 2011), Poland (2.8 in 2014), Albania (3.7 in 2011) and Croatia (4.8 in 2010) showed very high mean DMFT values [30, 31], which seems to indicate that important differences still exist among different European countries. In the last Spanish National Survey [16], the filling component of DMFT was much higher than the decay component for both ages, and this difference increased in group aged 15, where they found a mean DMFT of 1.34 and a filling component of 0.91. This finding was very similar to our results, with an increased filling component in group aged 15.

Lower SiC values for Galician schoolchildren were found, by comparing our results against those for other European regions [1, 7, 8]. Studies undertaken in other Spanish regions have shown a considerable increase in the restoration index in all cohorts across the period 1993–2000, showing RI of 52.6% at age 12 and 60.5% at 15-years-old. These indexes were higher in our study (73.29% at age 12 and 87.13% at age 15 years). In Galicia, the RI has increased from 20% in 1990 to 74% in 2010 for 12-year-old, evidence of the significant improvement in dental care levels [16]. If our results are compared to those of studies conducted in developing countries, it becomes clear that there is a widespread increase in decayed and missing teeth in these countries, which seems to indicate a worse oral health status, linked to poor access to dental attendance, and inadequate hygiene and food habits [9, 32].

In terms of the goals proposed by SESPO and the National Dental Association [13, 14], the goal for daily toothbrushing was not met at 12-years-old in our study population (88.3%), and the prevalence of caries-free teeth was 66.7 and 49.6% in 12- and 15-year-old, respectively, which is below the proposed goal. In contrast, the SiC goal was met in this sample (2.46 at 12-year-old); as well as the RI goal (73.28 and 87.13%, respectively), and the daily toothbrushing ≥90% at 15-years-old (94.8%). Spain has low caries levels, with DMFT values of 0.71 at age 12 and 1.34 at age 15 [16]. The DMFT/dmft in this study was 0.82 for the 12-year-old group and 1.38 at age 15, which indicates a similar oral health status in the Galician adolescents than in other Spanish areas. DMFT ≤1.0 set as the goal at 12-years-old, has already been achieved by this study population.

This study is not free of limitations. Firstly, the data refered to risk factors were obtained through a self-report questionnaire. Likewise, the significant associations found in this study between the caries prevalence and their determinants cannot be fully explained, considering that the current analysis is based on cross-sectional data, meaning that interpretation of results could be limited by the difficulty of discriminating between cause and effect. Another important limitation lies in not knowing the reason(s) why the patients surveyed chose to go to one or another health care service. Furthermore, comparison with other studies was limited by the definition of the variables of fluoride use and dietary habits, since the use of fluoride toothpaste and other fluoridated products has not been studied separately, and the definition of cariogenic diet has been fairly heterogeneous, with it being more appropriate to use objective data (such as grams of sugar intake) to measure this. Lastly, existing relationships with other risk factors previously described in the literature [1, 9, 18, 20, 21, 26] (socio-economic status, educational level, parents’ age and occupation, family income or anxiety about dental treatment) were not analysed. These circumstances are interesting, due to the possible interaction with the variables described in this study. The question about the age of starting toothbrushing could be difficult to answer/remember. We think this variable could be related in some extent to the presence of caries and dental plaque. In future studies it could be more reasonable to obtain that information from parents or caregivers. Further studies are thus called for.

## Conclusion

In conclusion, this study revealed that caries risk factors showed differences in schoolchildren aged 12- and 15 year-old. Both groups of age must be studied separately. Strongest evidence related to caries in 12-year-old group were found in frequency of toothbrushing and dental plaque. In 15-year-old group, electric toothbrush, time since the last visit to the dentist and type of dental care (public/private) had a stronger association with dental caries. Caries prevalence and mean DMFT/dmft increased from 12- to 15-year-old, in spite of improvement in oral hygiene at the age of 15. DMFT/dmft and caries prevalence was low according to WHO Oral Health Guidance for 2020, but there were inequalities among the population, and the goals proposed by the ACFF are still far to be reached.

## Additional files

Additional file 1: **Table S1.** Cohen’s Kappa concordance index between each of the five work teams and the external calibrator. (DOCX 17 kb)

Additional file 2: **Table S2.** Cohen’s Kappa concordance index between each of the five work teams. (DOCX 17 kb)

## Data Availability

The datasets used and analysed during the current study are available from the corresponding author on reasonable request.

## Abbreviations

ACFF: Alliance for a Cavity-Free Future
CI: Confidence Intervals
DMFT: Decayed, Missing, Filled teeth Index
DMFT/dmft: Decayed, Missing, Filled teeth (permanent + temporary) Index
OR: Odds Ratio
RI: Restoration Index
SD: Standard Deviation
SERGAS: Servizo Galego de Saúde / Galician Public Health Service
SESPO: Spanish Society of Epidemiology and Oral Public Health
SiC: Significant Caries Index
WHO: World Health Organization
