# Supplementary material for: Prevalence and caries-related risk factors in schoolchildren of 12- and 15-year-old: a cross-sectional study
Source: BMC Oral Health. 2019 Jun 18;19:120. doi: 10.1186/s12903-019-0806-5 (PMC6582601; doi:10.1186/s12903-019-0806-5)
Supplement: Supplementary file 1 — Table S1. Cohen’s Kappa concordance index between each of the five work teams and the external calibrator. (DOCX 17 kb) [file 12903_2019_806_MOESM1_ESM.docx]

Additional file 1: Table S1. Cohen's Kappa concordance index between each of the five work teams and an the external calibrator.

|  | **Unweighted Kappa** | |  | **Weighted Kappa** | |
| --- | --- | --- | --- | --- | --- |
| **Work teams** | **K_U_ (IC95%)** | **p-value** |  | **K_W_ (IC95%)** | **p-value** |
| **M** |  |  |  |  |  |
| Decayed teeth | 1 (1-1)^a^ | <0.001 |  | 1 (1-1)^a^ | <0.001 |
| Missing teeth^b^ | -- | -- |  | -- | -- |
| Filled teeth | 0.94 (0.93 - 0.96) | <0.001 |  | 0.97 (0.97 – 0.98) | <0.001 |
| **H** |  |  |  |  |  |
| Decayed teeth | 0.93 (0.91 – 0.95) | <0.001 |  | 0.97 (0.96 – 0.98) | <0.001 |
| Missing teeth^b^ | -- | -- |  | -- | -- |
| Filled teeth | 0.94 (0.92 – 0.96) | <0.001 |  | 0.97 (0.96 – 0.98) | <0.001 |
| **K** |  |  |  |  |  |
| Decayed teeth | 0.93 (0.91 – 0.95) | <0.001 |  | 0.97 (0.96 – 0.98) | <0.001 |
| Missing teeth^b^ | -- | -- |  | -- | -- |
| Filled teeth | 0.66 (0.62 – 0.69) | <0.001 |  | 0.65 (0.63 – 0.66) | <0.001 |
| **P** |  |  |  |  |  |
| Decayed teeth | 0.93 (0.91 – 0.95) | <0.001 |  | 0.97 (0.96 – 0.98) | <0.001 |
| Missing teeth^b^ | -- | -- |  | -- | -- |
| Filled teeth | 0.88 (0.86 – 0.90) | <0.001 |  | 0.82 (0.79 – 0.84) | <0.001 |
| **Z** |  |  |  |  |  |
| Decayed teeth | 1 (1-1)^a^ | <0.001 |  | 1 (1-1)^a^ | <0.001 |
| Missing teeth^b^ | -- | -- |  | -- | -- |
| Filled teeth | 0.81 (0.79 – 0.84) | <0.001 |  | 0.75 (0.73 – 0.77) | <0.001 |

^a^100% agreement.

^b^The Kappa index can not be computed because the students included in this concordance analysis had no missing teeth.
