# Supplementary material for: Prevalence and caries-related risk factors in schoolchildren of 12- and 15-year-old: a cross-sectional study
Source: BMC Oral Health. 2019 Jun 18;19:120. doi: 10.1186/s12903-019-0806-5 (PMC6582601; doi:10.1186/s12903-019-0806-5)
Supplement: Supplementary file 2 — Table S2. Cohen’s Kappa concordance index between each of the five work teams. (DOCX 17 kb) [file 12903_2019_806_MOESM2_ESM.docx]

Additional file 2: Table S2. Cohen's Kappa concordance index between each of the five work teams with.

|  | **Unweighted Kappa** | |  | **Weighted Kappa** | |
| --- | --- | --- | --- | --- | --- |
| **Work teams** | **K_U_ (IC95%)** | **p-value** |  | **K_W_ (IC95%)** | **p-value** |
| **M** |  |  |  |  |  |
| Decayed teeth | 0.87 (0.85 – 0.89) | <0.001 |  | 0.95 (0.94 – 0.96) | <0.001 |
| Missing teeth^a^ | -- | -- |  | -- | -- |
| Filled teeth | 0.79 (0.76 - 0.81) | <0.001 |  | 0.92 (0.91 – 0.93) | <0.001 |
| **H** |  |  |  |  |  |
| Decayed teeth | 0.82 (0.80 – 0.85) | <0.001 |  | 0.89 (0.87 – 0.91) | <0.001 |
| Missing teeth^a^ | -- | -- |  | -- | -- |
| Filled teeth | 0.76 (0.74 – 0.79) | <0.001 |  | 0.84 (0.82 – 0.86) | <0.001 |
| **K** |  |  |  |  |  |
| Decayed teeth | 0.67 (0.66 – 0.69) | <0.001 |  | 0.85 (0.83 – 0. 87) | <0.001 |
| Missing teeth^a^ | -- | -- |  | -- | -- |
| Filled teeth | 0.77 (0.74 – 0.79) | <0.001 |  | 0.91 (0.90 – 0.92) | <0.001 |
| **P** |  |  |  |  |  |
| Decayed teeth | 0.71 (0.68 – 0.75) | <0.001 |  | 0.90 (0.86 – 0.92) | <0.001 |
| Missing teeth^a^ | -- | -- |  | -- | -- |
| Filled teeth | 0.75 (0.73 – 0.78) | <0.001 |  | 0.92 (0.91 – 0.93) | <0.001 |
| **Z** |  |  |  |  |  |
| Decayed teeth | 0.73 (0.70 - 0.75) | <0.001 |  | 0.92 (0.91 – 0.93) | <0.001 |
| Missing teeth^a^ | -- | -- |  | -- | -- |
| Filled teeth | 0.81 (0.79 – 0.84) | <0.001 |  | 0.75 (0.73 – 0.77) | <0.001 |

^a^The Kappa index can not be computed because the students included in this concordance analysis had no missing teeth.
